# A global survey of healthcare professionals undertaking MRI of patients with cochlear implants: a heterogeneity of practice and opinions

**DOI:** 10.1259/bjr.20220213

**Published:** 2022-07-26

**Authors:** Rebecca Susan Dewey, Richard Bowtell, Padraig Kitterick

**Affiliations:** Sir Peter Mansfield Imaging Centre, School of Physics and Astronomy, University of Nottingham, Nottingham, UK; Hearing Sciences, Division of Mental Health and Clinical Neurosciences, School of Medicine, University of Nottingham, Nottingham, UK; National Institute for Health Research (NIHR) Nottingham Biomedical Research Centre, Nottingham University Hospitals NHS Trust, Nottingham, Nottingham, UK; Sir Peter Mansfield Imaging Centre, School of Physics and Astronomy, University of Nottingham, Nottingham, UK; National Acoustic Laboratories, Australian Hearing Hub, Macquarie University NSW 2109, Sydney, Australia

## Abstract

**Objective:**

To capture practice and opinions around the current clinical use of MRI in patients with cochlear implants (CIs), and to characterise patient progression from referral to image reporting.

**Methods:**

An online survey recruited 237 healthcare professionals between 9 December 2019 and 9 September 2020. Descriptive statistics and informal thematic analyses were conducted.

**Results:**

Respondents estimated that approximately 75% of CI users referred for an MRI proceeded to image acquisition, of which ~70% of cases comprised image acquisition on the head and the remaining cases on another area. They estimated that the proportion of these images that were usable was 93 and 99%, respectively. Confidence in most processes was high, with at least two-thirds of respondents reporting to be very or somewhat confident in obtaining consent and acquiring images. Conversely, fewer than half the respondents had the same confidence when splinting and bandaging the implant and troubleshooting any issues arising. Patient safety was rated of paramount importance, with patient comfort a clear second and image quality third.

**Conclusion:**

These findings highlight the need for consistent publication of clear, succinct, and standardised operating procedures for scanning patients with CIs and the requirement for regular training of radiographic and radiological healthcare professionals to address the heterogeneity of devices available.

**Advances in knowledge:**

There is a need to improve the communication to radiography and radiology personnel regarding the nature of CIs, the heterogeneity of devices in existence, and the key differences between them. CI users risk being underserved by diagnostic medical imaging.

## Introduction

MRI is the preferred diagnostic imaging technique providing high versatility, sensitivity, and specificity.^1^ A cochlear implant (CI) is indicated for severe and profound deafness and, consequent to improved identification of such hearing losses in neonates, is increasingly being administered in the first year of life.^2^

The implanted magnet and ferromagnetic material raise safety concerns around MRI of CI users due to the risk of severe discomfort, and ultimately implant magnet displacement.^3^ The resulting soft-tissue damage can require a prolonged period of healing, during which the CI cannot be used. Patients with CIs needing to undergo MRI have the option of surgical removal of the CI magnet to improve image quality nearer the implant or to facilitate imaging at higher field strength (*i.e.* 3 T). Surgical removal of the magnet comprises minor surgery with the potential for associated complications, resulting in a period without sound while the surgical wound heals [NEWREF_A]. Alternatively, a splint and bandage are applied to immobilise the implanted magnet. MR scanner gradients can induce unintended stimulation by the implant resulting in the perception of acoustic phenomena.^4–6^ Imaging of the head is confounded by substantial image distortions, even following magnet removal.^7,8^ Consequently, MRI may be avoided in favour of CT or positron emission tomography (PET). MRI is still the preferred imaging technique when serial (*e.g.* annual) re-assessment is required to monitor disease progression.^9^

A reported 33% of MRI scans of CI users resulted in complications^10^ despite at least 80% of those patients being fitted with the FDA-approved head wrap. Of these complications, 60% required additional surgical treatment and 40% could not complete the scan due to pain.^10^ Conversely, in vestibular schwannoma patients, only 14% of CI users experienced complications.^11^ A study spanning 14.5 years reported a complication rate of only 3.5% (including both CIs and auditory brainstem implants; ABIs).^12^ A search of the FDA MAUDE (Manufacturer and User Facility Device Experience) database^13^ reported 624 adverse events involving auditory implants (592 *in CIs*), including 384 magnet displacements, of which 59 were painful, and a further 48 incidents of pain without magnet displacement. Where compliance with manufacturer guidelines was noted, 37% of events occurred in cases where the guidelines had explicitly not been followed. A systematic review reported magnet dislocation in 11% of scans, and pain in 17% of scans, although the pain incurred by scanning with the magnet in place was described as still preferable to magnet removal.^13^

Manufacturers assign conditions on each CI model representing the suitability of the device for MRI. Some CIs are termed MR unsafe. CIs that can undergo MR are termed MR-conditional, explicitly meaning that they can only be scanned under certain conditions, including, but not exclusively: limiting the scanner magnetic field strength (in tesla, T), spatial gradient strength (tesla per unit distance, T m^−1^), and the amount of incident radiofrequency energy of sequences (specific absorption rate; SAR, in power per unit mass, W kg^−1^). Certain further procedures are also recommended for some CI models and scanner field strengths, *e.g.* the surgical removal of the internal retaining magnet, or the application of a splint and bandages. Such measures have been reviewed in detail and overlap somewhat with those of other active auditory implants.^13^

Three manufacturers currently have CIs on the market that are MR-conditional at 3.0 T. These devices contain rotating magnets that experience significantly less torque in a magnetic field. Such advances in implant technology have improved the practicality, safety, and comfort of MR scanning individuals with the newest generation of CIs, but this also significantly increases the heterogeneity of the MR compatibility/conditionality of CIs in circulation, as shown in Table 1. Every implant model has different associated safety conditions, and these can change.^14^ There is no single approach to conducting MR in CI users. Consequently, MRI departments need to keep up to date with the necessary safety advice, while also optimising image acquisition. Researching the different conditions for a given diagnostic MR question and a given model of implant takes time, and requires expertise and experience. Therefore, education in MR safety is paramount for managing these patients.

**Table 1.** A summary of the cochlear implant models implanted in the living population, together with the field strength at which they are MR conditional

CI manufacturer and model	MR unsafe	MR conditional	
		1.5 **T**	3 **T**
Cochlear CI612, CI622		✓	✓
Cochlear CI512, CI522, CI532, CI551		✓	
Cochlear CI422, CI24REH, CI24RE (CA), CI24RE (CS), CI24RE (ST)		✓	
Cochlear CI122M	✗		
Advanced Bionics HiRes Ultra		✓	✓
Advanced Bionics HiRes Ultra 3D		✓	✓
Advanced Bionics CLARION CI and CII	✗		
MED-EL SYNCHRONY CI		✓	✓
MED-EL CONCERTO, SONATA TI100, PULSAR CI100, C40+, C40		✓	
Oticon Neuro Zti 3T		✓	✓
Oticon Neuro Zti		✓	

CI, cochlear implant.

The primary objective of this study was to assess the “leaky pipeline” of patient progression through the system from referral to assessment. Secondary objectives were to characterise the decision-making process healthcare professionals undertake before deciding whether to scan a patient with a CI and what measures are required to ensure patient safety and optimise image acquisition. To achieve this, we conducted a global survey of healthcare professionals.

## Methods and materials

### Participants

Experimental procedures were approved by the London Fulham Research Ethics Committee (19/LO/1724). Participants gave informed consent online prior to participating. Participants were told that they could close the survey window at any point if they wanted to stop participating. Only completed survey responses were included in the sample. No identifying information was sought in the survey questions.

No formal sample size calculations were performed owing to the descriptive purpose of the study.

The study was advertised widely throughout professional bodies of radiographers, radiologists, and MR technologists and on social media between 9 December 2019 and 9 September 2020. 237 participants completed the survey.

### Survey design

The survey was designed by the research team in English. Questions were organised according to elements of the imaging pathway. Section 1 covered the country of origin, departmental funding, the respondent’s position, and available MR scanner field strengths. Section 2 covered the referral process for a CI user; appointment allocation, who makes decisions, and who scans. Section 3 addressed the appointment procedure; measures typically taken to prepare the patient for scanning (splinting, bandaging, etc.), adaptations to the scanning protocols, and the incidence of needing to pause the scan to administer patient care. Section 4 covered image quality. Section 5 asked the participant about their confidence completing each aspect of patient care.

To address the primary objective of the study, questions eliciting quantitative responses were constrained to integers. Where possible, all other questions used multiple choice responses to facilitate a quantitative descriptive analysis of the data. The final section used Likert scales comprising the options very confident; somewhat confident; neutral; somewhat lacking confidence; and very much lacking confidence. Respondents were asked to rank factors in order of importance. Finally, an open-ended question asked respondents to describe what for them is the most important issue related to scanning patients with CIs.

A survey draft was circulated in a consultation process with neighbouring Radiology departments. Following implementation of feedback from this consultation, the survey underwent peer review by a Reporting Radiographer, a Radiography Superintendent, and an MRI Clinical Scientist. At each stage, questions were added, removed, or amended to improve clarity. A pilot was conducted, which was successful, with minor alterations being made to correct errors or ambiguities. The survey was launched online using Jisc online surveys (onlinesurveys.ac.uk).

### Data processing and analysis

Responses were imported into SPSS v. 26 for data processing and inspection. Quantitative responses (the patient pipeline) were analysed using descriptive statistics. Multiple choice questions were analysed by summarising the percentage of respondents choosing each option. An informal thematic analysis was used on free-text responses, whereby themes were identified by visual inspection, and the frequency of theme occurrence was tallied.

## Results

### Demographics of the respondents

**Figure 1.**
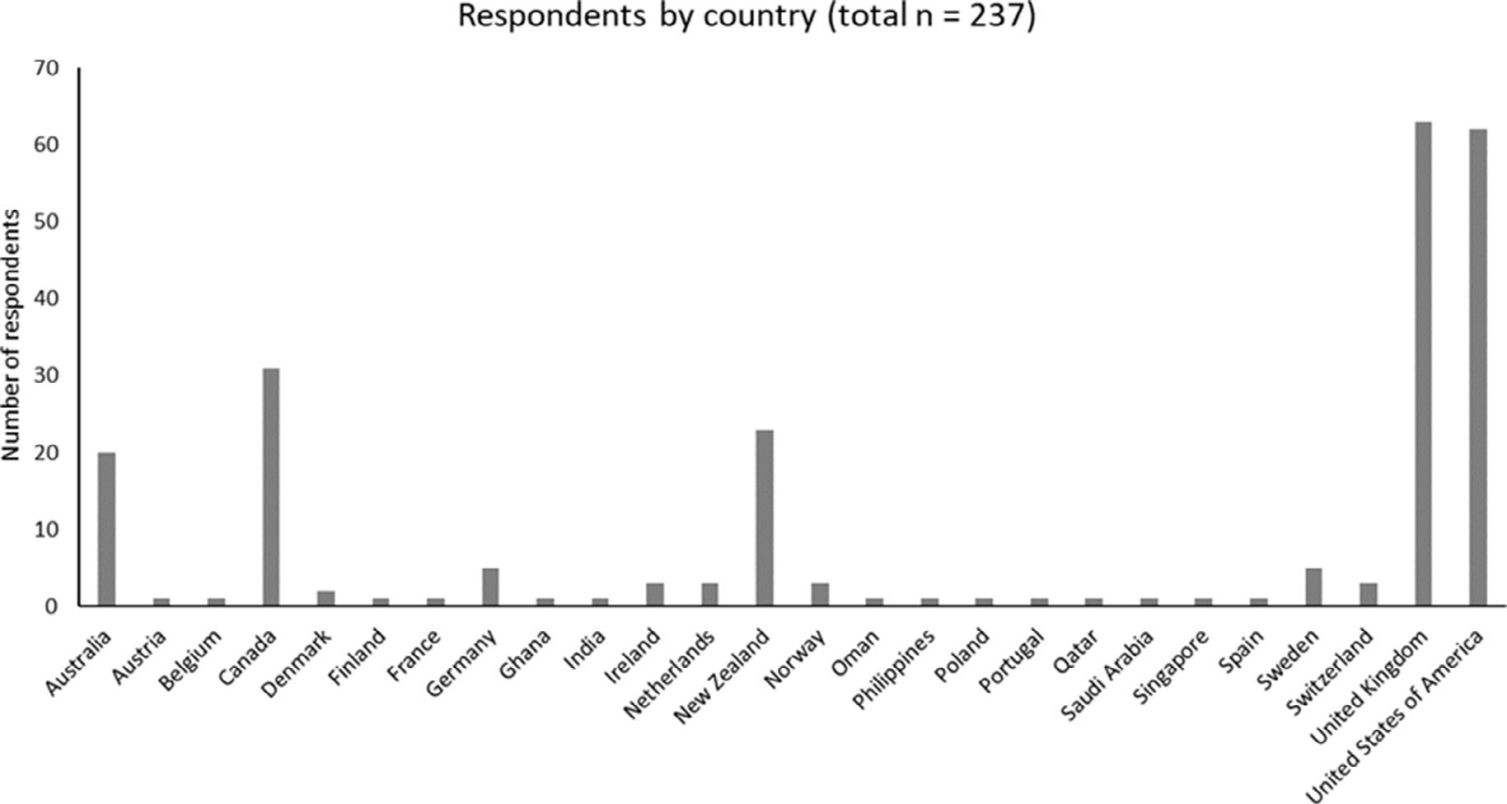
Location of respondents by country.

Figure 1 shows the geographical distribution of respondents across 26 countries. Participants reported their institutional funding to be 31% private, 31% public, 11% state, 6% trust, 21% multiple, other, or declined to answer. Respondents were 39% senior radiographers, 21% radiographers, 23% superintendent radiographers, 4% consultants, 2% managers, 1% junior doctors, 1% trainees, and 10% in other positions. 95% of respondents had access to a 1.5 T MR scanner, 65% of respondents had access 3.0 T, 3% had access to 7.0 T, and 3% had access to scanners at 1.0 T and lower. Employment duration within the sample ranged from less than a year to more than 15 years, with the modal duration being “more than 15 years”.

### Internal procedures around scanning patients with CIs

#### The decision to scan or not

Radiographers contributed to making the decision whether or not to scan in 16% of the departments, with senior radiographers at 30% and superintendent radiographers at 29%, consultants 35%, registrars 3%, house officers and junior doctors 10%, managers 10%, nurses 1% and an additional 29% of sites answering other/don’t know/decline to answer. 19% reported that CI users in their department were always scanned by the same member of staff, whereas 65% of respondents reported the opposite (the remaining 16% selected other/don’t know/decline to answer).

#### The field strength to scan at

Most (87%) respondents would consider scanning a CI user at 1.5 T, with very small numbers favouring lower field strengths (8%). 10% would scan at 3.0 T, but none would consider scanning higher field strengths. 6% of respondents said they would not scan a CI user at any field strength.

#### Resources for decision making

Table 2 shows the resources used in deciding whether to scan, and how to improve image quality, and the rate at which those resources were available to respondents. Resources mentioned included in-house physics or safety specialists and/or ENT radiologists, surgical or audiological specialists, the MRI Safety Reference Manual by Frank Shellock^15^ or the associated MRIsafety.com website, seeking advice or training from other hospitals with more experience, and the MagResource website. For improving image quality, participants said they would also consult an MR physicist or MR applications specialist. To each of these questions, there were approximately five respondents who reiterated that they would not scan a patient with a CI at their site.

**Table 2.** Resources that respondents reported preferring to use, and which are available to them, for assisting with the decision whether or not to scan, and assisting with improving image quality, in patients with a CI

Resource	Decision whether to scan		Improve image quality	
	Would use	Have available	Would use	Have available
Online manufacturer resources	92%	89%	70%	72%
In-house protocol	70%	73%	51%	65%
Online MR physics resources	40%	48%	46%	48%
Ask a colleague	38%	46%	55%	59%
Peer-reviewed literature	26%	23%	33%	27%
Own judgement	22%	35%	38%	47%
Textbooks	4%	18%	13%	20%
Social media	3%	12%	9%	13%
Other	16%	10%	8%	7%

CI, cochlear implant.

#### Additional safety measures

Table 3 shows which measures respondents considered to facilitate MR scanning a patient with a CI. Measures that were given under “other” comprised asking the patient what they had experienced previously, moving the bed very slowly into the scanner, or immediate image review by a radiologist to ensure that the patient is not in the scanner for any longer than necessary. 25 respondents (11%) stated that they had never, or would never, scan a patient with a CI. Only 86 respondents (36%) reported needing to stop the scan due to the patient experiencing discomfort, of which, 15% reported this happening more often when scanning the head, and 15% reported it to be more common when scanning an area outside the head (“below the neck”). Having paused scanning, respondents reported taking additional measures prior to resuming scanning (Table 2 final column). Individual responses comprised talking to or reassuring the patient, adjusting the bandage or splint, or allowing the patient a break. 15 respondents (17%) said that they were unable to resume the scan.

**Table 3.** Measures respondents would consider taking or actually had taken to facilitate scanning a patient with a CI

Measure	Would consider taking	Had taken prior to scanning	Taken to resume scanning (% of those who paused)
Modifying scanner protocol	62%	53%	91%
Bandage around head	53%	47%	37%
Manufacturer’s splint	43%	38%	30%
In-house splint	25%	23%	14%
Place patient on bed outside magnet hall	43%	36%	n/a
Modify the way they position the patient’s head	38%	32%	n/a
Modify the position of the patient’s head for scanning	35%	30%	37%
Modify scanner hardware selection	21%	19%	19%
Sedation or general anaesthetic	10%	8%	8%
Local anaesthetic	8%	6%	3%
Other	17%	19%	26%

CI, cochlear implant.

Data represent the rate of respondents agreeing as a percentage of the number of respondents. The final column gives the percentage of the 86 respondents who had stopped a scan to administer additional measures.

### The leaky pipeline from referral to image interpretation

**Figure 2.**
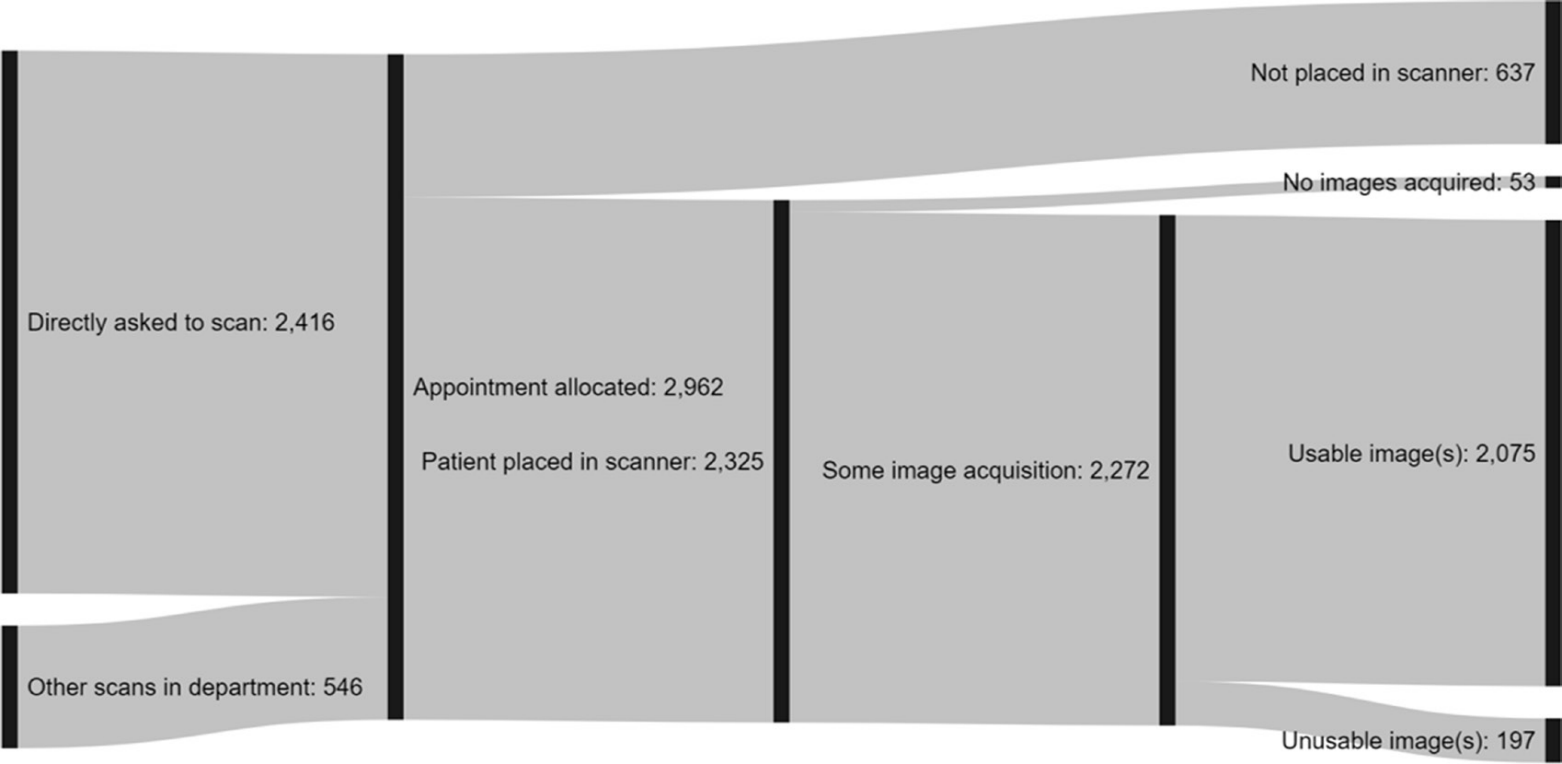
The “leaky pipeline” of patients progressing from referral through to usable images. The pipeline shows absolute numbers of patients at each stage. Not included in this figure was also a question asking the respondent for the number of acquisitions they managed to complete. This was reported to be 2110 overall (93% of those who completed some image acquisition). The pattern was very similar when patient numbers were split between scans of the head, and of another area than the head (“below the neck”), with a breakdown of 1560 for the head (97% of those who completed some image acquisition), and 667 for the body (92% of those who completed some image acquisition). The only notable difference being that for patients undergoing a head scan had a higher proportion of those allocated appointments were never placed in the scanner, whereas for patients being scanned below the neck, a higher proportion of patients placed in the scanner had no image acquisition performed.

**Figure 3.**
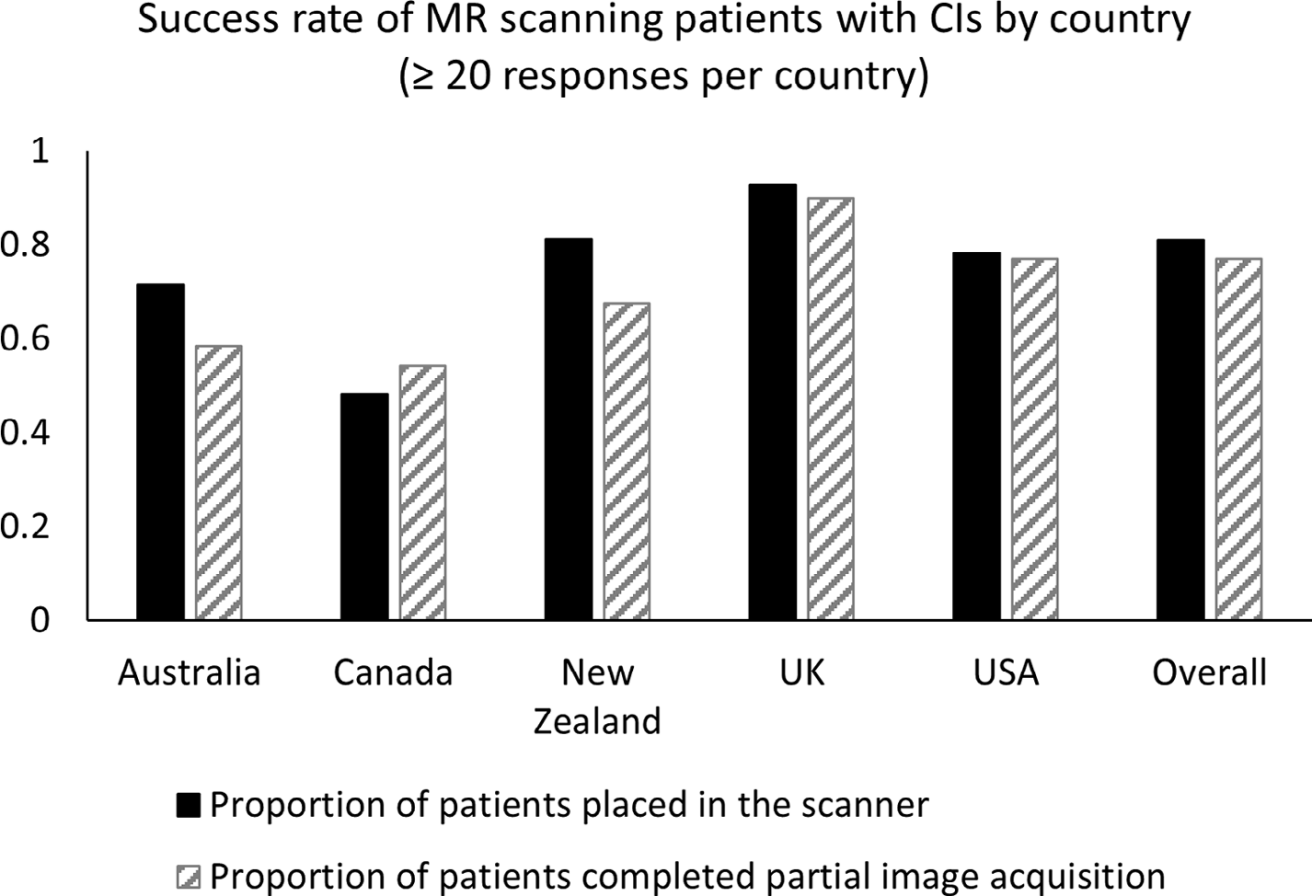
Proportions of patients reported to have reportedly been allocated appointments who went on to be successfully placed in the scanner and have some images acquired, by country (countries with at least 20 respondents). CI, cochlear implant.

Figure 2 shows the numbers of patients that respondents estimated their departments have been asked to scan, allocated appointments, placed in the scanner, acquired some images, and ultimately produced usable images. The visual pattern of the pipeline was very similar between scans of the head, and below the neck. The highest level of attrition occurred between the allocation of an appointment and the patient being placed in the scanner. Figure 3 shows an analysis by respondent country of the proportions of patients reported to have reportedly been allocated appointments who went on to be successfully placed in the scanner and have some images acquired. This was conducted for the countries with at least 20 respondents each, namely Australia, Canada, New Zealand, the UK and the USA, highlighting the bias toward English-speaking countries in the sample.

### Respondent opinions

**Figure 4.**
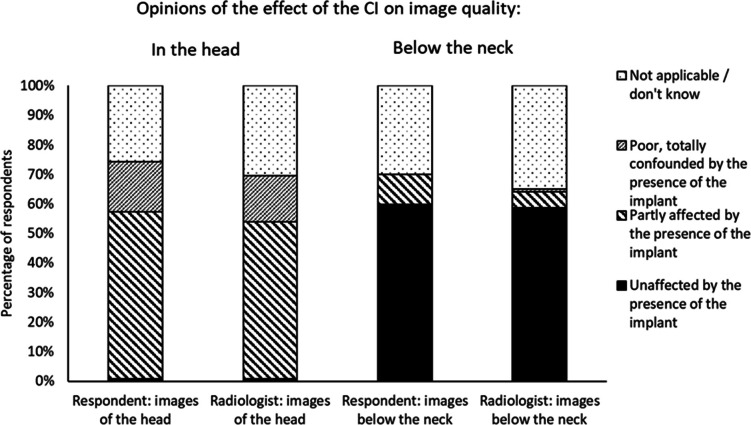
Healthcare professional opinions on the degree to which MR images are affected by the presence of a CI, separated by area scanned. CI, cochlear implant.

Figure 4 summarises answers to questions about the extent to which image quality is affected by the presence of a CI. In order to differentiate between the differing expectations of the two professional groups, respondents were asked to first give their own opinion and then subsequently their assumption about the opinion of the radiologist (although the sample did include a small number of radiologists, who would likely have given the same answers for both of the sets of questions). Overall, respondents stated that radiologists were less optimistic about image quality than they were.

**Figure 5.**
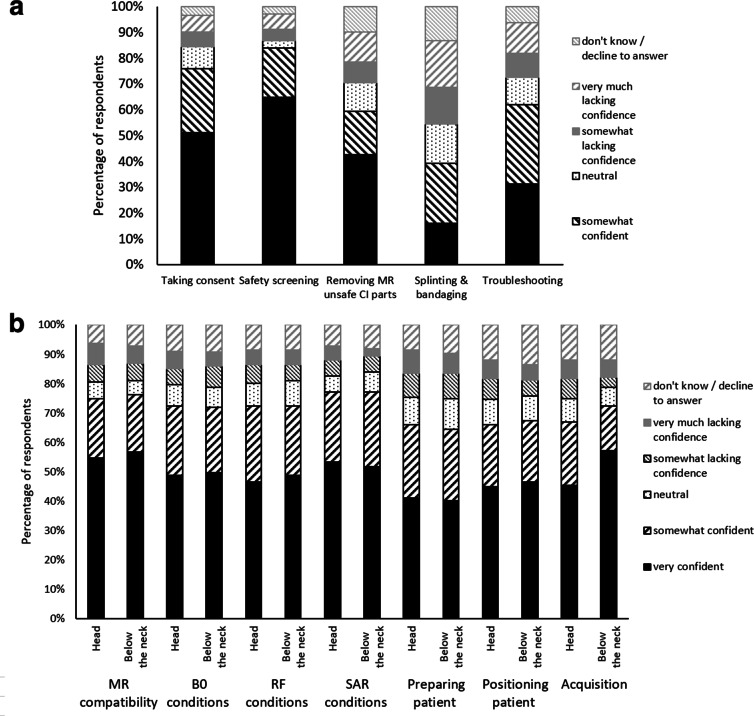
Confidence in performing tasks related to scanning a patient with an MR scanner, as rated by respondents, divided into (**A**) tasks that apply to all patients with CIs regardless of the area being scanned, and (**B**) tasks that are specific to the area of the body being scanned, and thus responses were given separately for scans of the head and of an area of the body other than the head (“below the neck”). CI, cochlear implant; SAR, specific absorption rate.

Figure 5a and b show the degree to which respondents had confidence in their ability to conduct each element of scanning a patient with a CI. Confidence in performing these tasks varied, with high confidence in consenting and screening patients, and considerably lower confidence when handling the CI and troubleshooting any issues arising with the patient.

### Priorities and issues moving forwards

**Figure 6.**
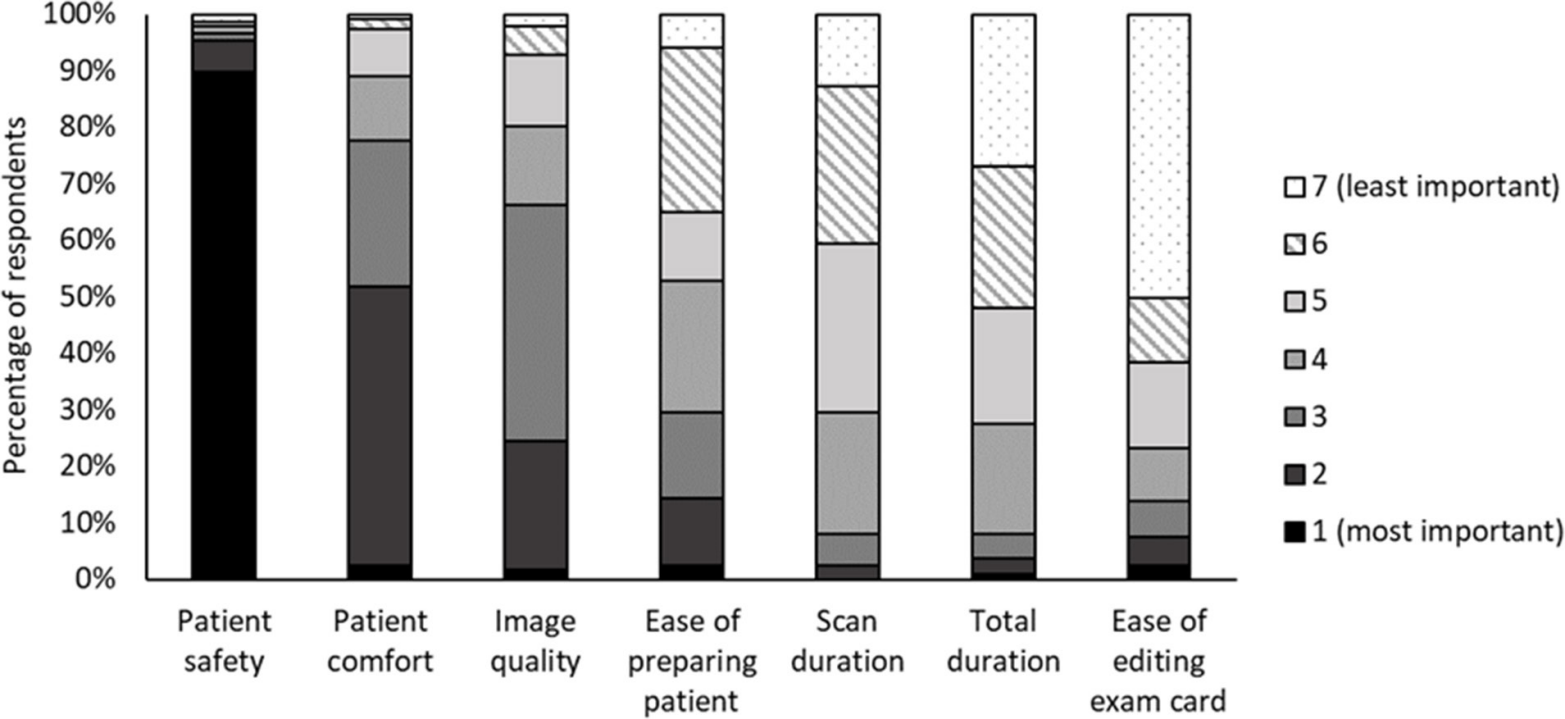
Ratings of relative importance of various factors around ease and convenience of scanning patients with CIs as scored by respondents to the questionnaire, where black signifies the highest importance (a score of “1”) and white signifies the lowest importance (a score of “7”). 90% of respondents rated patient safety as being the most important factor. CI, cochlear implant.

Figure 6 shows the relative importance of factors associated with the process of MR scanning a patient with a CI. Patient safety was rated of paramount importance, with patient comfort a clear second and image quality coming third. Ease of editing the exam card was viewed as the least important factor.

**Figure 7.**
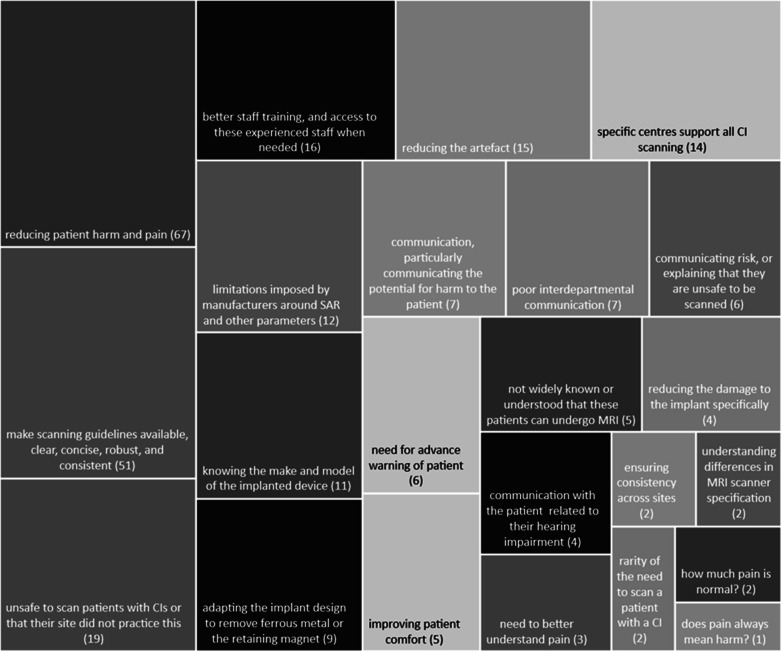
A visual representation of responses to the question “What do you think is the most important issue related to scanning patients with Cochlear Implants, that research needs to address?”, where the size of the box represents the number of times the theme appears in the responses. The most common theme was reducing patient harm and pain occurring 67 times.

Respondents were asked what they thought was the most important issue with regards to scanning patients with CIs. A visual representation of these responses is shown in Figure 7. Of primary concern was the need for improvements in the MR compatibility of devices, reducing patient harm and pain, reducing the artefact, the limitations imposed by manufacturers around SAR and other parameters, adapting the implant design to remove ferrous metal or the retaining magnet, improving patient comfort, and reducing the damage to the implant specifically.

Next most-frequently mentioned was the need for scanning guidelines to be more available, clear, concise, robust, and consistent across manufacturers, ensuring that the make and model of the implanted device is known so that the correct guidelines can be obtained, or that manufacturer guidelines are implemented consistently across sites. Some respondents expressed a need for better staff training, and access to these experienced staff or facilities when needed due to the rarity of CI user referrals. Some expressed a need to address the risk of scanning versus the benefit of the procedure.

Respondents emphasised communication issues, particularly communicating the potential for harm to the patient, the risk of scanning, or explaining to a patient that they are unsafe to be scanned, as well as concerns around communication related to the patient’s hearing impairment. Some participants expressed the need for advance warning and having time to prepare for scanning the patient, and specifically that interdepartmental communication within the hospital made this challenging. Finally, there was a wish to better understand the mechanisms behind the pain the patient experiences, and to receive some guidance on what is considered a normal or safe level of discomfort.

## Discussion

This study presents the healthcare professional opinions around the MRI of a patient who has one or more CIs. The literature contains many case reports communicating success or failure of attempts to conduct MRI in this group, including reports of magnet displacement despite all reasonable precautions being taken.^16–20^ As advances in CI design improve the safety of undertaking such scans, attention turns to improving the quality of images acquired in such patients.^21^ There is no consensus on the safety procedures needed for scanning patients with CIs, and a recent review article highlights the heterogeneity of advice provided by manufacturers, and the resulting variation in the degree of success even when such advice is followed.^22^ A recent article reporting the results of a survey of CI patients revealed less than 10% of the cohort to have undergone MRI since implantation, and 70% of those scans resulting in a complication of some sort.^23^ There has not been a global survey conducted of healthcare professionals, and this is the first as such to provide a snapshot of procedures and beliefs within the MR/ENT community. Further, it would be useful in future studies, to identify what role the referring physicians play in this process and what could be improved upon moving forward.

Our primary aim was to determine whether there are specific points along the pathway from clinical referral through to image acquisition and interpretation that “leak” patients. The highest attrition seems to occur between the allocation of an appointment and the patient being placed in the scanner. Responses suggest this may be due to departmental policy not to scan CI users, or a widespread belief that there is no safe way to scan these patients. The pattern was similar for scans of the head, and below the neck, with only a couple of notable differences. The first exception was that for patients undergoing a head scan a higher proportion of those allocated appointments were never placed in the scanner, which may be related to departmental policies based around the belief that there is no safe way to scan patients with CIs, and that image quality will be poor. Conversely, a higher proportion of patients placed in the scanner for acquisition below the neck had no images acquired. This could be due to the strong torque experienced by the implanted retaining magnet when in the fringe field of the magnet causing unanticipated discomfort for the patient. The data also suggest that 70% of CI users needing MRI were due to undergo head MRI, with the predictable consequence in a reduction in the number of these images being clinically useful, likely due to CI artefacts. Therefore, while the presence of a CI does not appear to lead to the widespread avoidance of scanning, image quality remains a significant limiting factor when imaging these patients.

One of the secondary objectives of this study was to characterise what safety measures are taken and how standard image acquisitions are adapted for use. Availability of the necessary resources may well be an issue, with only 73% of respondents reporting having access to in-house protocols for scanning CI users. Useful good practice highlighted in the responses included having a radiologist present during scanning to view patient images immediately such that the patient need not stay in the scanner any longer than necessary, asking the patient what measures had facilitated a successful scan for them on previous occasions, and offering continuous reassurance and updates on progress throughout the scanning process. It was unfortunately beyond the scope of this study to determine which factors lead to higher confidence in scanning patients with CIs, thus decreasing the group of patients that could have been scanned. Identifying these factors is the next step toward clearer recommendations and training for clinical professionals.

This study did not capture numbers of complications, making it difficult to compare directly with previously conducted studies. It is now necessary to investigate what measures, sources of expertise, assistance, or information, or availability of resources are needed to address the shortcomings highlighted in the present article. For example, the task that reported the greatest spread in confidence levels was that of splinting and bandaging patients; but it was not established what respondents felt they were lacking access to. The sampling strategy was not cross-sectional, and the survey was advertised as pertaining to MRI of patients with CIs, which may have introduced recruitment bias by deterring staff working at sites that do not scan CI users at all. Further, while a small number of survey respondents did participate from non-English-speaking countries, this was a minority of participants. Finally, the present article does not address the problem of patients not even reaching the referral stage for receiving an MRI scan; *i.e*. patients who never enter the pipeline because MRI is disregarded at the outset by the patient’s clinical care team.

## Conclusion

In a global survey of 237 people conducted in English, respondents reported a total of 2962 referrals of CI users. Of these, 76% completed image acquisition on the head and 78% below the neck, with 89 and 91% of patients successfully scanned having some usable images being acquired in the head and below-the-neck, respectively. Confidence in obtaining consent and performing image acquisition was generally high. Conversely, respondents were much less confident with handling the CI, preparing the patient for scanning, and troubleshooting any issues arising. Patient safety was rated of paramount importance by the cohort, with patient comfort a clear second and image quality coming third. The results from this survey highlight the need for consistent publication of standardised operating procedures for scanning patients with CIs and potentially for regular training of radiographic and radiological healthcare professionals on the vast array of devices in use.
